# From dysconnectivity to symptoms: large-scale resting-state networks relate to psychotic phenomenology in schizophrenia

**DOI:** 10.1186/s12888-026-08282-9

**Published:** 2026-07-21

**Authors:** Ting Yu, Shaokun Zhao, Yanli Li, Na Li, MengZhuang Gou, WenJin Chen, XiaoYing Wang, JingHui Tong, Yunlong Tan

**Affiliations:** Beijing Huilongguan Hospital, Capital medical university, Peking University HuiLongGuan Clinical Medical School, Beijing, P. R. China

**Keywords:** Schizophrenia, Resting-state functional connectivity, Visual-default mode network decoupling, Hallucinations, Symptom modeling

## Abstract

**Background:**

Schizophrenia is increasingly conceptualized as a disorder of large-scale brain network integration, yet how specific symptom phenotypes relate to reproducible resting-state functional connectivity (rsFC) signatures remains unclear.

**Methods:**

Resting-state fMRI data from 386 patients with schizophrenia and 212 healthy controls were analyzed to characterize large-scale functional connectivity patterns. Group-level connectivity differences were first identified (uncorrected *P* < 0.05, for exploratory feature selection), followed by within-patient analyses examining associations between altered connectivity and symptom subitems while adjusting for demographic factors. Multivariate models were then used to evaluate whether connectivity patterns showed systematic associations with individual symptom profiles.

**Results:**

Group comparisons revealed a dysconnectivity pattern characterized by reduced cross-network coupling between the visual network and higher-order systems, alongside selective increases in frontoparietal circuits. Within patients, connectivity alterations showed distinct association patterns across delusion, hallucination, and negative-symptom subitems. Multivariate analyses further indicated modest associations with several hallucination (e.g., H8, R²≈0.09) and delusion (e.g., D3, R²≈0.07) subitems, whereas associations with negative symptoms were minimal and showed limited generalization.

**Conclusions:**

These findings support a hierarchical dysconnectivity profile centered on impaired perceptual–cognitive integration and suggest that specific positive-symptom phenotypes may be associated with partially consistent rsFC signatures. Overall effect sizes were modest, indicating that rsFC captures only a limited component of symptom variability.

**Clinical trial number:**

Not applicable.

## Background

Schizophrenia is a severe psychiatric disorder characterized by heterogeneous symptom dimensions spanning hallucinations, delusions and negative symptoms [1]. Accumulating evidence increasingly supports the view that schizophrenia arises from disrupted communication among distributed brain networks rather than isolated regional abnormalities, consistent with dysconnection models that emphasize aberrant functional integration [2]. At rest, functional brain networks—including the default mode network (DMN), salience network (SLN), frontoparietal/central executive systems and sensory networks—form coordinated architectures that support internally oriented mentation, salience detection and cognitive control [3]. Disruptions across these systems have been repeatedly observed in schizophrenia, yet the specific network mechanisms underlying distinct symptom dimensions remain incompletely understood.

Two major gaps currently limit progress in the field. First, although numerous studies have reported case–control abnormalities in resting-state functional connectivity (rsFC), the direction and spatial distribution of these findings remain inconsistent across cohorts and analytic pipelines. For instance, investigations in early-stage psychosis have demonstrated broadly reduced between-network coupling in patients relative to controls after stringent correction, yet also revealed increased interindividual variability in attention-related networks, suggesting that both mean connectivity shifts and network instability may characterize illness expression [4]. Second, symptom mapping typically relies on total or composite scores, which may obscure finer-grained, mechanistically relevant symptom phenotypes. Prior studies indicate that certain symptom expressions align with specific network dysfunctions—for example, aberrant SLN–DMN communication has been associated with hallucinations and other reality-distortion phenomena [5]. 

To address these limitations, the present study aimed to link large-scale dysconnectivity patterns with specific symptom dimensions at the subitem level. We analyzed Resting-state functional MRI (rs-fMRI) data from 386 patients with schizophrenia and 212 healthy controls, while controlling for demographic covariates. Using ENIGMA-atlas–based ROI [6, 7] time series, we first identified rsFC edges showing group-level differences, then examined within-patient associations between these altered connections and individual symptom subitems using partial correlations (adjusting for age and education). Finally, we implemented multi-output partial least squares regression (PLSR) models to examine multivariate associations between rsFC patterns and multiple symptom subitems. We hypothesized that schizophrenia would exhibit preferential disruption of perceptual–association integration—particularly reduced coupling between the visual and higher-order networks—and that positive-symptom subtypes (hallucinations and delusions) would display more stable and more consistent rsFC signatures than negative symptoms.

## Method

### Participants and clinical assessment

#### Recruitment and grouping

A total of 212 healthy controls (HC) and 386 patients with schizophrenia (SZ) were enrolled. Patients were enrolled during their first hospitalization at Beijing HuiLongGuan Hospital. Schizophrenia diagnosis was established by two independent and experienced psychiatrists using the International Classification of Diseases-10 (ICD-10) criteria [8]. The inclusion criteria for patients with first-episode schizophrenia were as follows: (1) age of 16–45 years, (2) total illness duration of < 3 years. Exclusion criteria were as follows: suffer from other severe mental disorders evaluated by experienced psychiatrists or by SCID, with the presence of intellectual disability, neurological or uncontrolled medical conditions, recent treatment with immunotherapy or hormone therapy, pregnancy/lactation, alcohol or substance use disorder. Healthy controls were recruited through advertisements with the inclusion criteria as follows: (1) age of 16–45 years, (2) no diagnoses of current or history of mental illness, and (3) no history of psychosis in their first-degree relatives. Other exclusion criteria were same as those for the SZ group.

All participants provided written informed consent. The study was approved by the Ethics Committee of Beijing Hui-Long-Guan Hospital and conducted in accordance with the Declaration of Helsinki.

#### Demographic and clinical assessments

The demographic information collected for all participants included age, sex, and years of education. Symptom severity in the SZ group was comprehensively assessed using the Positive and Negative Syndrome Scale (PANSS), which consists of three domains: positive symptoms, negative symptoms, and general psychopathology. The negative-symptom subscale (N1-N7) was further used in subsequent functional connectivity analyses to examine its associations with large-scale brain network organization [9]. 

Hallucination and delusion symptoms were assessed using the corresponding subscales of the Psychotic Symptom Rating Scales (PSYRATS) [10]. The PSYRATS provides detailed, multidimensional evaluations of psychotic experiences, capturing phenomenological aspects such as frequency, duration, distress, conviction, and functional impact. Specifically, hallucinations were quantified using the PSYRATS-H (Hallucination Subscale), while delusional symptoms were evaluated using the PSYRATS-D (Delusion Subscale) to characterize belief strength, preoccupation, and associated distress.

Negative symptoms were assessed using the PANSS negative-symptom items (N1-N7), which were further incorporated into the predictive modeling analyses.

### MRI acquisition and preprocessing

#### MRI acquisition

All MRI scans were performed using a SIEMENS MAGNETOM Prisma scanner (syngo MR D13D). T1-weighted images were acquired using a magnetization-prepared rapid gradient-echo sequence (MPRAGE) with the following parameters: TA = 6:38, PAT = 2, voxel size = 0.8 × 0.8 × 0.8 mm³, FoV = 256 mm, TR = 2400 ms, TE = 2.22 ms, TI = 1000 ms, flip angle = 8°.

rs-fMRI was acquired with: TA = 5:46, voxel size = 2.0 × 2.0 × 2.0 mm³, 72 slices, FoV = 208 mm, TR = 800 ms, TE = 37 ms, flip angle = 52°, multiband factor = 8. A total of 420 volumes were acquired for each participant, resulting in a scan duration of approximately 5 min and 36 s. The acquisition protocol and scan duration were identical across all participants.

#### Preprocessing

Preprocessing was conducted using fMRIPrep v20.2.1 [11]. The first 10 volumes were discarded to allow for signal equilibration. Functional images were motion-corrected (MCFLIRT), nonlinearly normalized to MNI152 space, and resampled to 3-mm isotropic resolution to improve computational efficiency and inter-subject registration stability. Spatial smoothing (6-mm FWHM Gaussian kernel) was applied prior to ROI time series extraction to enhance signal-to-noise ratio and reduce residual anatomical variability across subjects, consistent with standard fMRIPrep recommendations. Nuisance regression included Friston 24 motion parameters, white matter and cerebrospinal fluid signals, and linear drift terms. Temporal filtering was performed using a band-pass filter (0.01–0.1 Hz). Participants exceeding 3 mm translation or 3° rotation were excluded. Quality control was performed by reviewing fMRIPrep-generated reports. Given that subsequent analyses were conducted at the ROI level (ENIGMA atlas), resampling is unlikely to materially affect ROI-averaged signals.

#### Functional connectivity

Time series were extracted from ROIs defined by the ENIGMA atlas [6]. Pearson correlation coefficients were computed between each pair of ROI time series using the GRETNA toolbox [12] to construct whole-brain functional connectivity (FC) matrices, and subsequently transformed to Fisher’s z-scores. Analyses focused on connections within and between key large-scale networks, including the DMN, AN, SLN, and FPN.

### Statistical analyses

The clinical measures described above provided clearly defined predictor and outcome variables for subsequent analyses, including partial correlation analyses and multi-output partial least squares regression (PLSR).

#### Demographic analyses

Group differences in sex were examined using chi-square tests, while age and years of education were compared using independent-sample t-tests in IBM SPSS Statistics. Significant demographic variables were included as covariates in subsequent models.

#### Between-group FC comparisons

Edge-wise independent-sample t-tests were conducted to identify FC differences between SZ and HC groups by using IBM SPSS Statistics. Group comparisons were conducted using an uncorrected threshold of *P* < 0.05 for exploratory feature selection.

#### Symptom-network associations

Partial correlations were performed within the SZ group to evaluate associations between symptom subitems and FC edges that showed significant group differences (*p* < 0.05), adjusting for age and education. Analyses were implemented in Python (Ver 3.13.5). Significant associations were visualized through network-level diagrams.

#### Predicting symptoms using PLSR

To assess whether FC profiles could jointly predict multiple symptom manifestations, multi-output PLSR models were constructed. PLSR is well suited for high-dimensional neuroimaging data and addresses multicollinearity by projecting predictors onto a low-dimensional latent space. Multi-output PLSR analyses were implemented in Python (Ver 3.13.5) using the scikit-learn package.

Predictor variables included standardized FC edges and demographic covariates with significant group differences. Outcome variables comprised standardized scores of symptoms subitems significantly associated with FC.

All variables were centered and scaled before modeling. Sample sizes were: 346 for the delusion model, 267 for the hallucination model, and 369 for the negative-symptom model.

An 80/20 train–test split (random_state = 42) was used to evaluate generalization performance, with five-fold cross-validation applied within the training set. Five-fold cross-validation determined the optimal number of latent components by maximizing mean R² across outputs. Final models were fitted on the training set and evaluated using R² on the independent test set. Standardized regression coefficients were used to identify FC predictors with the strongest contributions. These coefficients were interpreted as indicators of relative contribution within the latent variable space, accounting for the multicollinearity of the functional connectivity features. By projecting predictors into an orthogonal latent space, PLSR provides stable weights that represent each edge’s contribution to the symptom dimensions within the context of the entire network profile.

## Result

### Demographic and clinical characteristics

A total of 212 HC and 386 patients with SZ were included in the final analysis. Group differences in demographic characteristics were examined using independent-sample t-tests and *χ²* tests, as summarized in Table 1. SZ patients were significantly younger than HC participants (*t* = 7.539, *p* < 0.001) and had fewer years of education (*t* = 2.282, *p* = 0.023). No significant sex difference was observed between groups. Given these findings, age and years of education were included as covariates in subsequent functional connectivity analyses.

**Table 1:** Comparison of demographic characteristics between the HC and SZ groups

Variables	HC(*n* = 212)	SZ(*n* = 386)	*t*/χ²	*p*
Sex (male/female)	107/105	185/201	0.448	0.503
Age (M ± SD)	35.61 ± 11.63	28.42 ± 10.17	7.539	< 0.001***
Years of education (M ± SD)	13.72 ± 2.78	13.13 ± 3.36	2.282	0.023*

Note: M ± SD denotes mean ± standard deviation. Sex was analyzed using the χ² test, while age and years of education were examined using independent-sample t-tests. *p* < 0.05; ***p < 0.001

### Between-group differences in functional networks

**Fig. 1:**
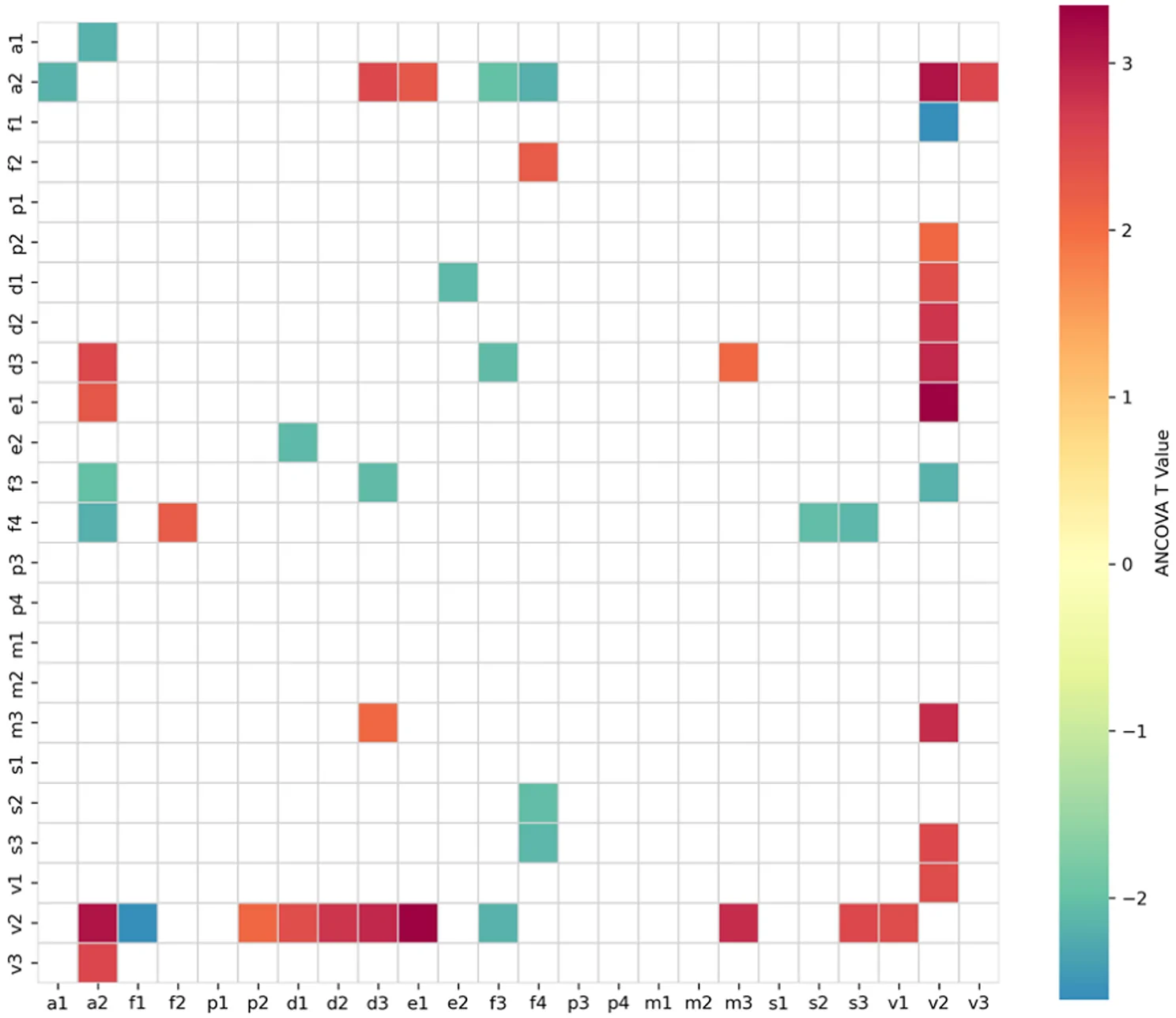
Between-group comparison of functional networks. Note: This figure displays 23 FC edges with significant group differences (*p* < 0.05; see Table 2). Warm colors indicate connections stronger in Healthy Controls (HC > SZ). Cool colors denote connections stronger in Schizophrenia Patients (SZ > HC)

Based on two-sample comparisons (uncorrected *P* < 0.05), a total of 23 FC edges showed exploratory group differences (Fig. 1; Table 2). Fourteen edges were stronger in the HC group than in the SZ group, predominantly involving cross-network coupling between the visual network (VN) and the DMN, auditory network (AN), executive control network (ECN), and sensorimotor network (SMN). Examples include ECN-VN (e1-v2, *t* = 3.35), AN-VN (a2-v2, *t* = 3.09), DMN-VN (d3-v2, *t* = 2.91), and SMN-VN (m3-v2, *t* = 2.85). These patterns suggest relatively preserved perceptual-cognitive integration in HC.

Conversely, nine edges were stronger in the SZ group. These primarily involved frontoparietal network (FPN) pathways and salience/visual couplings, such as FPN-VN (f1-v2, *t* = -2.61), AN-FPN (a2-f4, *t* = -2.20), FPN-VN (f3-v2, *t* = -2.18), and FPN-SLN (f4-s3, *t* = -2.10). These increases may reflect frontal hyperconnectivity or compensatory engagement in SZ, occurring alongside reduced perceptual-cognitive integration.

Overall, the group-difference pattern reveals a consistent hierarchical profile: SZ patients exhibit widespread reductions in VN-DMN/AN/ECN/SMN integration, accompanied by selectively increased connectivity in frontal-dominant circuits (e.g., FPN with SLN/AN).

**Table 2:** Functional network differences between the HC and SZ groups

Connection	*t*	*p*	Connectivity strength
e1-v2	3.35	< 0.001	HC > SZ
a2-v2	3.09	0.002	HC > SZ
d3-v2	2.91	0.004	HC > SZ
m3-v2	2.85	0.005	HC > SZ
d2-v2	2.76	0.006	HC > SZ
f1-v2	-2.61	0.010	SZ > HC
a2-v3	2.55	0.011	HC > SZ
a2-d3	2.51	0.013	HC > SZ
s3-v2	2.51	0.013	HC > SZ
v1-v2	2.48	0.014	HC > SZ
d1-v2	2.43	0.016	HC > SZ
a2-e1	2.31	0.022	HC > SZ
f2-f4	2.26	0.025	HC > SZ
a2-f4	-2.2	0.028	SZ > HC
a1-a2	-2.18	0.030	SZ > HC
f3-v2	-2.18	0.030	SZ > HC
f4-s3	-2.1	0.037	SZ > HC
d1-e2	-2.09	0.037	SZ > HC
p2-v2	2.09	0.038	HC > SZ
d3-f3	-2.08	0.039	SZ > HC
d3-m3	2.07	0.039	HC > SZ
f4-s2	-2.05	0.041	SZ > HC
a2-f3	-2.03	0.044	SZ > HC

Note: only connections significant at *p* < 0.05 are listed. Direction specifies the group with the significantly higher connectivity strength for that edge (e.g., HC > SZ means Healthy Controls had significantly stronger connectivity than Schizophrenia patients)

### Symptom-network associations

Exploratory partial correlation analyses were conducted within the SZ group to evaluate associations between symptom subitems and FC edges that showed group differences, adjusting for age and education. Based on the FC edges showing significant group differences, partial correlation analyses were conducted within the SZ group while controlling for age and years of education. These analyses examined the association patterns between individual symptom subitems and the predefined FC sets.

**Fig. 2:**
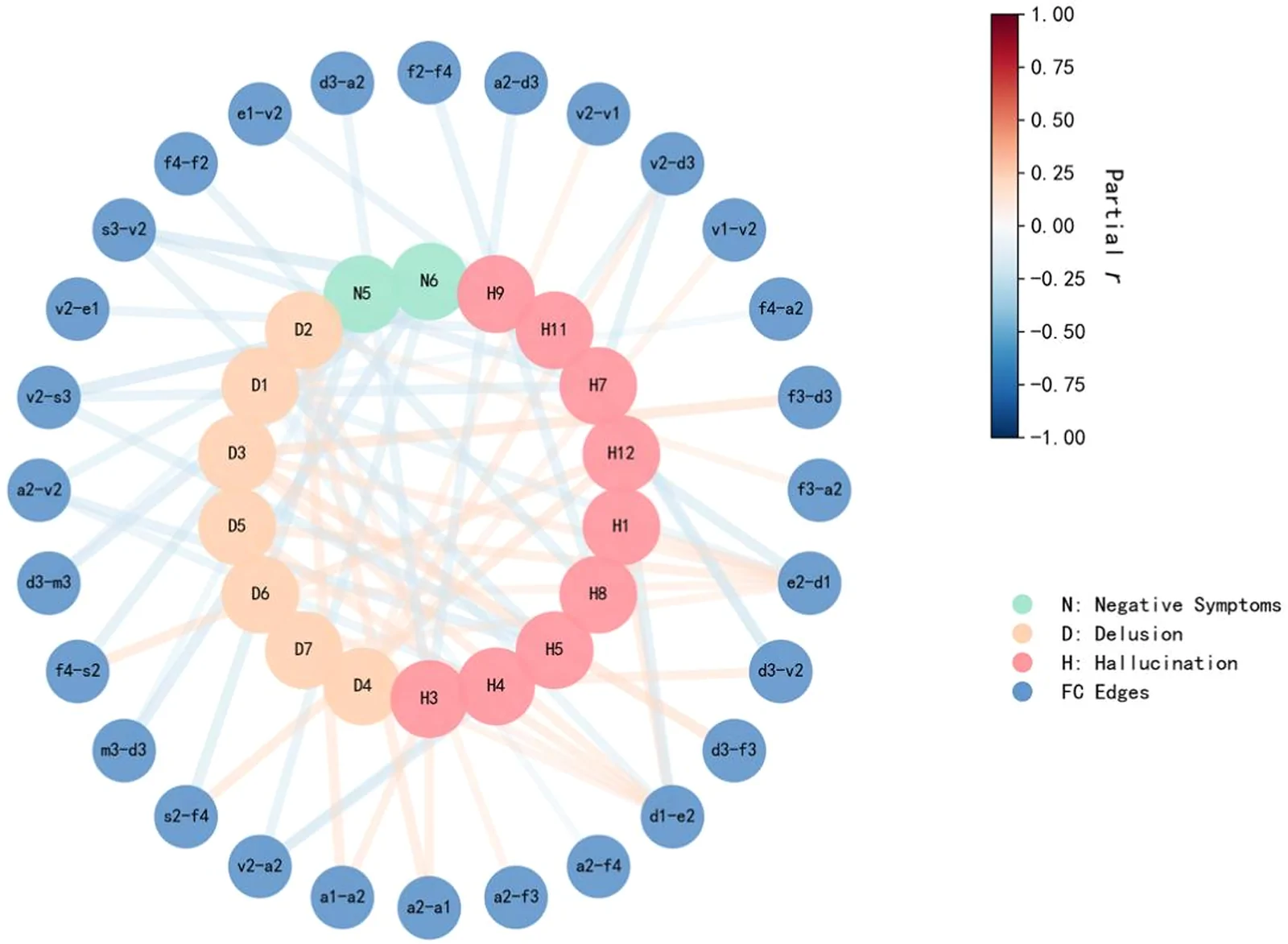
Partial correlation results between symptoms and FC edges. Note: This figure displays FC edges with significant partial correlations (p < 0.05; see Table 3) with symptom sub-items across Delusion, Hallucination, and Negative Symptom domains. Warm colors indicate a positive correlation (r > 0), where higher symptom severity is associated with stronger connectivity. Cool colors indicate a negative correlation (r < 0), where higher severity is associated with weaker connectivity

**Table 3:** Partial correlation results between symptoms and FC edges

Symptom	Symptom Sub-item	Connection	Partial *r*	*p*
Delusion	D1	a1-a2	0.193	0.012
Delusion	D4	d1-e2	0.187	0.015
Delusion	D3	d3-v2	0.182	0.019
Delusion	D2	d1-e2	0.18	0.02
Delusion	D5	d1-e2	0.163	0.035
Delusion	D1	a2-f3	0.162	0.036
Delusion	D3	v1-v2	0.162	0.037
Delusion	D6	d1-e2	0.159	0.038
Hallucination	H8	s3-v2	-0.223	0.012
Hallucination	H6	d3-v2	-0.205	0.021
Hallucination	H11	d1-e2	-0.203	0.023
Hallucination	H4	a2-v2	-0.202	0.023
Hallucination	H4	s3-v2	-0.199	0.026
Hallucination	H7	f2-f4	-0.199	0.026
Hallucination	H11	f4-s2	0.198	0.027
Hallucination	H6	s3-v2	-0.193	0.031
Hallucination	H2	a2-d3	-0.186	0.037
Hallucination	H10	e1-v2	-0.184	0.039
Hallucination	H3	a2-v2	-0.179	0.045
Hallucination	H10	d3-v2	-0.177	0.048
Hallucination	H2	a1-a2	0.177	0.048
Negative_Symp	N6	d3-m3	-0.212	0.004
Negative_Symp	N5	f4-s2	-0.204	0.006
Negative_Symp	N6	a2-v2	-0.189	0.01
Negative_Symp	N5	m3-d3	-0.157	0.034

Note: D for Delusion (Positive Symptoms), H for Hallucination (Positive Symptoms), and N for Negative Symptoms. FC Edge identifies the brain connection (Node1-Node2). only results (*p* < 0.05) are included

The partial correlation analysis revealed dimension-specific and spatially distributed FC association patterns across different symptom domains (Table 3; Fig. 2).

For delusions, significant associations clustered primarily within cross-network connections involving frontal, limbic, and parieto-occipital pathways. Subitems such as D2, D1, and D4 showed moderate positive correlations with edges including d3-f3, a1-a2, and d1-e2 (Partial *r* = 0.16–0.21). Notably, some edges (e.g., D3-v2-d3, D-d1-e2) exhibited negative correlations, suggesting that delusions may engage both enhanced and diminished coupling mechanisms.

For hallucinations, associations were predominantly negative and involved pathways related to parietal-visual integration, frontal regions, and multisensory processing. Subitems such as H8, H6, and H4 showed significant negative correlations with edges like s3-v2, d3-v2, or a2-v2 (Partial *r* = -0.18 to -0.22), indicating potential decoupling in multimodal sensory networks. A few positive correlations (e.g., H11-f4-s2) suggest the presence of compensatory or symptom-specific network recruitment.

For negative symptoms, significant associations involved reductions in motor-visual and frontoparietal integrative pathways. N6 showed strong negative correlations with d3-m3 and a2-v2 (Partial *r* = -0.19 to -0.21), and N5 showed a similar pattern with f4-s2. These findings support the hypothesis that negative symptoms are related to impaired cross-network integrative functioning.

Taken together, each symptom dimension displayed unique yet partially overlapping FC association signatures: delusions were characterized by frontal-dominant cross-network increases, hallucinations by sensory integration decoupling, and negative symptoms by reduced motor-visual and integrative pathways.

### Prediction of symptoms from functional connectivity

**Fig. 3:**
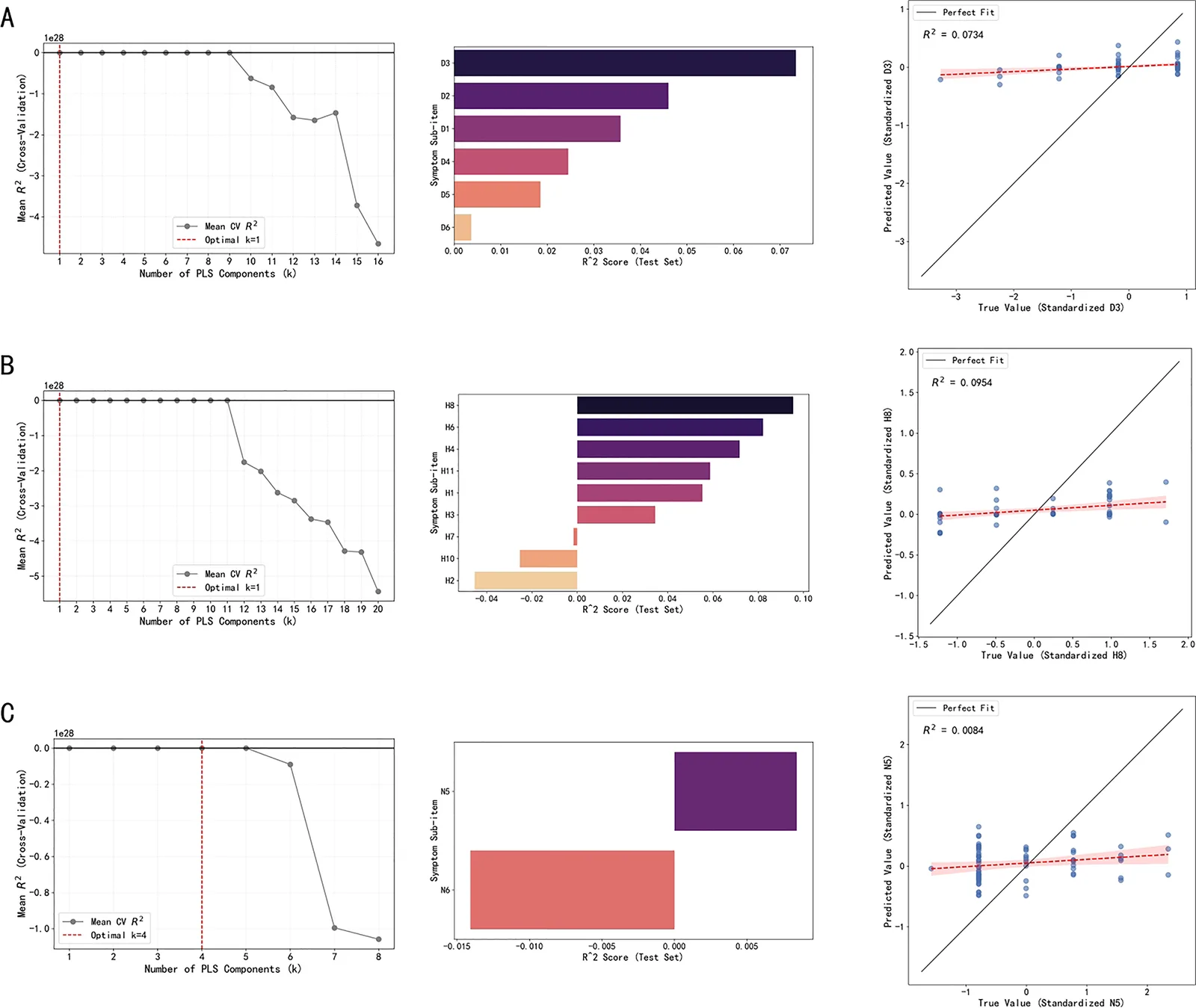
Prediction of symptoms using multi-output PLSR. Note: This figure adopts a 3 × 3 layout to showcase the model performance of the Multi-Output PLSR model for the three primary symptom categories: (**A**) Delusion, (**B**) Hallucination, and (**C**) Negative Symptoms. Each row corresponds to a specific symptom category and sequentially displays: (Left) The cross-validated R^2^ curve as a function of the optimal number of latent variables (k); (Middle) A bar chart showing the R^2^ values for all sub-items within that category, calculated on an independent test set; and (Right) A scatter plot depicting the standardized true (observed) values versus the predicted values for the sub-item demonstrating the best predictive performance (R^2^) within that category

Three independent PLSR models were constructed using FC edges that showed significant group differences as predictors, evaluating the predictability of delusion, hallucination, and negative-symptom subitems (Fig. 3; Table 4). Overall, the models yielded limited but detectable effects for hallucinations and delusions, whereas prediction of negative symptoms remained minimal. These predictive analyses were conducted without correction for multiple comparisons across symptom subitems and should be interpreted as exploratory.

For hallucinations, the model showed relatively stronger performance compared with other symptom domains, although overall effects remained limited. The optimal number of latent components was k = 1 (mean cross-validated *R²* = -0.0356). The negative cross-validated R² indicates that model performance did not generalize reliably during training, suggesting limited predictive stability. On the independent test set, several hallucination subitems showed limited but statistically detectable. The item with the highest R² was H8 (argumentative or commentary hallucinations), with an *R²* of 0.0954, indicating a modest effect size. Other subitems included H6 (command hallucinations, *R²* = 0.0821), H4 (somatic hallucinations, *R²* = 0.0718), and H11 (*R²* = 0.0587). These results suggest that certain hallucination subitems show relatively consistent FC patterns detectable by the model. Coefficient analyses indicated that edges such as a2-d3, e2-d1, and f4-s2 contributed most strongly across multiple hallucination subitems.

For delusions, model stability improved after removing potentially aberrant FC edges, and some modest effects were observed. Feature removal was performed in a data-driven manner within the training set only, based on the identification of extreme or unstable model weights. The optimal component number remained *k* = 1 (mean cross-validated *R²* = -0.0875). On the test set, D3 showed the highest *R²* (0.0734), followed by D2 (*R²* = 0.0460) and D1 (*R²* = 0.0357). Coefficient results suggested that a2-f3, a1-a2, and d3-f3 contributed most strongly to the model.

In contrast, associations with negative symptoms were limited. Although the optimal number of components was *k* = 4 (mean cross-validated *R²* = 0.0174), test-set performance showed minimal generalization capacity. The two key negative-symptom subitems had *R²* values near zero: N5 (motor retardation) showed *R²* = 0.0084, and N6 (blunted affect) showed *R²* = -0.0141. These findings indicate that FC patterns explained minimal variance in negative symptoms, possibly due to the multidimensional and heterogeneous nature of negative symptoms exceeding what resting-state FC can capture.

Collectively, these results indicate that FC patterns showed modest associations with positive symptoms such as hallucinations and delusions, whereas associations with negative symptoms were minimal. These effects were modest and should be interpreted cautiously given the limited variance explained and negative cross-validated performance.

**Table 4:** Prediction results of symptom subitems using multi-output PLSR

Symptom	Symptom Sub-item	Optimal *k*	*R* ^2^
Hallucination	H8	1	0.0954
	H6	1	0.0821
	H4	1	0.0718
	H11	1	0.0587
	H	1	0.0553
	H3	1	0.0344
	H7	1	-0.0017
	H10	1	-0.0253
	H2	1	-0.0454
	---	---	---
Delusion	D3	1	0.0734
	D2	1	0.046
	D1	1	0.0357
	D4	1	0.0245
	D5	1	0.0185
	D6	1	0.0037
	---	---	---
Negative Symptoms	N5	4	0.0084
	N6	4	-0.0141

Note: Optimal *k* is the best number of latent variables used in the PLSR model for that symptom group, determined via cross-validation. *R*^*2*^ represents the coefficient of determination, quantifying the proportion of variance in the symptom sub-item score explained by model estimates

## Discussion

This study yields three primary insights into the network basis of schizophrenia.

First, we identified a hierarchical dysconnectivity profile characterized by reduced cross-network coupling between the VN and higher-order systems (DMN, AN, ECN, and SMN), accompanied by selectively increased connectivity within frontoparietal-dominant circuits.

Second, symptom–network associations within patients were subitem-specific, underscoring that distinct positive- and negative-symptom components engage partially dissociable network configurations rather than a uniform “positive-symptom axis.”

Third, multivariate analyses indicated that rsFC features showed modest associations with several hallucination and delusion subitems, whereas associations with negative symptoms remained negligible—highlighting domain-specific tractability for connectivity-based biomarkers.

At a broader level, our group-difference pattern aligns with previous reports showing that healthy controls exhibit stronger between-network coupling than schizophrenia patients, especially across sensory–association interfaces [4]. However, prior work also highlights illness-stage effects and greater temporal variability in patients’ connectivity, particularly within attention-related systems [4]. Such heterogeneity may help explain inconsistencies across rsFC studies—schizophrenia likely involves both mean connectivity shifts and increased dynamical instability, with detectability contingent on cohort characteristics, preprocessing choices, and analytical granularity (edge-wise vs. network-averaged measures).

Our findings further converge with the triple-network framework implicating altered interactions among the DMN, SLN, and executive systems as central to psychopathology [3]. The SLN serves as a key integrator that detects salient stimuli and mediates transitions between internally and externally oriented cognition. Abnormal SLN–DMN coupling has been repeatedly linked to hallucinations and reality distortion, and our results extend this model by highlighting disrupted communication between sensory (particularly visual) and associative systems as a central feature of schizophrenia.

A particularly salient feature of our results is the marked reduction in VN coupling with multiple higher-order networks. This pattern suggests that schizophrenia may involve impaired integration of sensory input with internal models and executive control processes, resulting in noisy or biased perceptual inference. Within the predictive-processing framework, the observed VN–DMN decoupling can be interpreted as a neural signature of impaired hierarchical inference. From a neuroanatomical perspective, this communication breakdown may reflect reduced coupling between visual cortical regions and medial prefrontal areas (mPFC), a core hub of the DMN. This interpretation is inferential and based on network-level connectivity patterns rather than direct ROI-to-ROI findings. Within a predictive-processing framework, weakened feedforward signaling of visual prediction errors may fail to update higher-order priors in associative cortices, while aberrant top-down influences from DMN regions such as the mPFC may contribute to exaggerated or misattributed percepts. This imbalance between sensory evidence and prior expectation provides a mechanistic basis for hallucinations and delusional beliefs, linking large-scale network dysconnectivity to computational disturbances in predictive error signaling [13]. 

From a clinical neuropsychological perspective, the subitem-level differentiation provides insight into the cognitive mechanisms of psychotic experiences. For instance, PSYRATS-H8 (commentary or argumentative hallucinations) involves verbal self-monitoring and inner-speech attribution processes, implicating aberrant coupling between auditory–salience and visual–default mode systems. In contrast, PSYRATS-H4 (somatic hallucinations) likely engages multisensory integration circuits, consistent with the observed parietal–visual decoupling. Similarly, delusion subitems such as D3 (“being controlled” beliefs) may reflect impaired predictive modeling of self-generated actions within frontoparietal–limbic loops, aligning with the notion of disrupted self-agency signaling [14]. These distinctions suggest that symptom subitems are not merely psychometric artifacts but correspond to dissociable perceptual–cognitive network dysfunctions. Consistent with this conceptualization, hallucination-related subitems showed relatively stronger effects in our PLSR analyses. Although effects were modest, they indicate that resting-state FC captures a measurable but partial component of symptom variance. This modest explanatory power likely reflects the multifactorial origins of symptoms—arising from interactions among distributed neural systems, environmental context, and measurement variability. Nonetheless, the identification of reproducible, subitem-level rsFC signatures represents a meaningful step toward dimensional, precision phenotyping of psychosis [15]. 

In contrast, negative symptoms demonstrated poor generalization in multivariate models. This may reflect their multidimensional nature (e.g., avolition, blunted affect, psychomotor slowing) and dependence on broad, distributed circuits not well captured by static rsFC measures. Task-based paradigms, dynamic connectivity metrics, or multimodal integration (e.g., structural and neurochemical data) may be required to capture these complex phenomena. Additionally, secondary factors such as medication effects, depressive symptoms, and social withdrawal can further obscure rsFC–symptom relationships [16, 17]. 

Notably, our findings partially converge with those reported by large consortia such as ENIGMA-SZ and B-SNIP, which have identified widespread reductions in sensory–association connectivity and variable frontoparietal hyperconnectivity across cohorts [7, 18, 19]. However, the present study extends these observations by linking network-level dysconnectivity to fine-grained symptom subitems and by examining individual-level model generalization—an approach rarely implemented in consortium-scale analyses. Future cross-cohort harmonization with ENIGMA pipelines could further validate these subitem-level neural signatures.

It is noteworthy that some cross-validated R² values were negative, indicating limited generalizability of the models during training. This suggests that the observed effects should be interpreted cautiously and may partly reflect sample-specific patterns rather than robust predictive relationships [20, 21]. 

Several limitations should be acknowledged. First, group-difference edges were identified at an uncorrected threshold (*P* < 0.05), which increases the likelihood of false positives; future studies should apply more stringent network-level correction. Second, although the PLSR models achieved statistical significance, the explained variance (R^2^ 0.03–0.09) was modest, suggesting that rsFC alone provides limited individual-level explanatory power and should be integrated with multimodal data in the future. Third, residual confounding (e.g., medication dosage, illness duration heterogeneity) may still influence connectivity–symptom relationships despite statistical adjustment [22]. In addition, residual group differences in head motion may still exist, particularly in schizophrenia populations, which represents a limitation of the present study. Finally, rsFC is sensitive to motion and preprocessing pipelines; while a standardized fMRIPrep workflow was adopted, future replication across alternative denoising and dynamic approaches would enhance robustness [23]. In addition, non-neuronal factors such as vascular-related signal fluctuations or potential occipital signal bleed may influence resting-state connectivity estimates, particularly in regions adjacent to major vascular structures (e.g., circle of Willis), and should be considered when interpreting visual-network findings.

Despite these limitations, the present findings advance a hierarchical model of schizophrenia centered on disrupted perceptual–cognitive integration. Our results demonstrate that specific positive-symptom dimensions are partially associated with distributed rsFC features, providing a framework for future biomarker development and individualized symptom monitoring. Integrating network-level mapping with dimensional modeling approaches may ultimately facilitate mechanistically informed, personalized interventions in psychosis.

## Conclusion

In a large rs-fMRI cohort, schizophrenia showed a coherent dysconnectivity profile characterized by reduced visual-to-association network integration and selective increases in frontoparietal-dominant connectivity. Symptom-subitem analyses revealed differentiated rsFC signatures across delusions, hallucinations, and negative symptoms, and multivariate models showed modest associations with select positive-symptom subitems but not negative symptoms. These findings emphasize the importance of hierarchical, cross-network integration for psychotic phenomenology and highlight both the promise and current limits of rsFC-based symptom modeling.

## Data Availability

The data associated with this study have not been deposited in any publicly available repository however, they are available from the corresponding author upon request.

## Code availability

The custom analysis scripts supporting the findings of this study are available from the corresponding author upon reasonable request.

## Abbreviations

AN: Auditory network
DMN: Default mode network
ECN: Executive control network
FPN: Frontoparietal network
FC: Functional connectivity
rsFC: resting-state functional connectivity
HC: Healthy controls
ICD-10: International Classification of Diseases-10
MPRAGE: Magnetization-prepared rapid gradient-echo sequence
mPFC: medial prefrontal cortex
MCFLIRT: Motion correction
PLSR: Partial least squares regression
PANSS: Positive and Negative Syndrome Scale
PSYRATS: Psychotic Symptom Rating Scales
rs-fMRI: Resting-state functional MRI
SLN: Salience network
SZ: Schizophrenia
SMN: Sensorimotor network
SCID: Structured Clinical Interview for DSM-IV-TR Axis-I Disorders
VN: Visual network
